# Field Suppression of Spotted Wing Drosophila (SWD) (*Drosophila suzukii* Matsumura) Using the Sterile Insect Technique (SIT)

**DOI:** 10.3390/insects13040328

**Published:** 2022-03-26

**Authors:** Rafael A. Homem, Zeus Mateos-Fierro, Rory Jones, Daniel Gilbert, Andrew R. Mckemey, Glen Slade, Michelle T. Fountain

**Affiliations:** 1 BigSis, 5 Weighbridge Row, Cardiff Road, Reading RG1 8LX, UK; zeus@bigsis.tech (Z.M.-F.); dan@bigsis.tech (D.G.); glen@bigsis.tech (G.S.); 2 NIAB EMR, New Road, East Malling, Kent ME19 6BJ, UK; r.jones26@live.co.uk (R.J.); michelle.fountain@niab.com (M.T.F.); 3 Berry Garden Growers, Tatlingbury Oast, Tonbridge, Kent TN12 6RG, UK; 4 Independent Co., Ltd., Oxford OX2 9AF, UK; andy.mckemey@gmail.com

**Keywords:** biodiversity, mark–release–recapture, integrated pest management, raspberry, strawberry, cherry, crop pest, fertility, longevity, sustainability

## Abstract

**Simple Summary:**

The spotted wing drosophila (SWD) (*Drosophila suzukii*) is an economically important insect pest that can cause significant damage to a wide range of soft and stone fruit. Growers currently use chemical insecticides and labour-intensive cultural management to reduce the economic impact of SWD. The sterile insect technique (SIT) is a chemical free, environmentally friendly and proven method of insect control: sterile males are released into a specific area, where they mate with wild females, which then produce no, or very few offspring. This study aimed to demonstrate, for the first time, the use of SIT to control SWD in field conditions without physical barriers that could significantly prevent invasion of wild SWD or escape of sterile males. Trials were carried out in open polytunnels, which are known to be extremely susceptible to invading SWD in contrast to glasshouse settings, where the enclosure minimises SWD pressure. Male SWD were made 99% sterile using X-rays and released twice per week into a 7.2 ha site growing everbearing strawberries. The method achieved season-long control with suppression of up to 91% compared with two untreated control sites. We also investigated how the wild SWD population varied throughout the season and the longevity of the released sterile male SWD. This study suggests that SIT may be a very effective, environmentally sustainable alternative to chemical insecticides for the control of SWD in commercial conditions. It also provided insights to help optimise the practical deployment of the tool.

**Abstract:**

*Drosophila suzukii* (spotted wing drosophila—SWD) is an economically important pest of soft and stone fruit worldwide. Control relies on broad-spectrum insecticides, which are neither fully effective nor environmentally sustainable. The sterile insect technique (SIT) is a proven, effective and environmentally friendly pest-management tool. Here, we investigated, for the first time, the potential of using SIT to control *D. suzukii* in field conditions without physical barriers that limit insect invasion. A proprietary method of rearing and irradiation with X-rays was used to obtain males that were > 99% sterile. Sterile males were released twice per week from April to October 2021 on a site in Kent, UK, where everbearing strawberries were grown in open polytunnels. The infestation of wild female *D. suzukii* was monitored weekly using red sticky traps with dry lure at the treated site and at two similar control sites that did not receive sterile male releases. Releases of sterile males suppressed the wild female *D. suzukii* population by up to 91% in comparison with the control sites. We thus demonstrated the feasibility of SIT to achieve season-long control of *D. suzukii* using early, sustained and dynamically targeted releases of sterile males. This provides a promising environmentally friendly method to control this important pest.

## 1. Introduction

*Drosophila suzukii* (Matsumura) (Diptera: Drosophilidae) is an economically important pest that originated in Southeast Asia and has recently spread globally. It reached North America (the USA and Canada) and Europe, including Spain and Italy, in 2008 and was first detected in the UK in 2012 [1,2,3]. Unlike the vast majority of *Drosophila* species, *D. suzukii* has the ability to oviposit in a wide range of ripening soft and stone fruits (e.g., strawberry and cherry), due to a morphologically modified serrated ovipositor which allows eggs to be laid into ripening fruit [4]. Eggs hatch into larvae, which develop inside the fruit, rendering produce unmarketable, hence causing significant economic losses [5]. Oviposition wounds also provide entry points for secondary infections by microorganisms and other insects, thus causing even greater economic damage to fruit crops [6,7]. For example, in three USA states, losses of more than USD 500 million in 2008 were attributed to *D. suzukii* [1].

Currently, *D. suzukii* control is reliant on the application of insecticides alongside cultural control methods, in particular, more frequent harvesting, enhanced hygiene and the use of insect exclusion nets [8]. The limited number of approved chemical active ingredients, the potential for regulatory restrictions on some of these, and the threat of insecticide resistance, pose a threat to *D. suzukii* control. Recently, spinosad resistance has been detected in wild *D. suzukii* populations in the USA [9]. Effective control strategies, less reliant on insecticide inputs, are therefore needed to achieve integrated pest management (IPM).

The sterile insect technique (SIT) is an environmentally friendly pest management tool that has been applied to control several pests of economic importance [10]. Although the concept of using sterile males to control natural insect populations was independently proposed by different researchers [11,12,13], it was Knipling [13]—encouraged by the pioneering work of G. A. Runner [14] and H. J. Muller [15]—who proposed ionising radiation could induce sexual sterility in males of the new world screwworm fly, *Cochliomyia hominivorax* (Coquerel), a highly destructive livestock pest [16]. Bushland and Hopkins [17] further optimised radiation doses and conditions in laboratory experiments and the world’s first SIT field trial took place on two small islands (Sanibel and Captiva Island), off the west coast of Florida, USA in 1953 [18].

Since then, SIT has been used against dozens of species and on six continents, both for the suppression and the eradication of pest species [19]. Recent laboratory-based studies have provided encouraging results regarding the potential feasibility of using SIT as a tool for *D. suzukii* control. Research covering optimised mass-rearing systems [20], determination of optimal radiation doses for sterility [21,22] and the impact of radiation on *D. suzukii* fitness [23,24] have been published. In addition, a great deal is already known on the biology and behaviour of *D. suzukii*. Understanding the biology and ecology of the pest can enable the best uses of SIT for *D. suzukii* control.

The lifespan of *D. suzukii* in laboratory conditions is around 20–60 days and it differs greatly across studies with some reports showing that flies can survive up to 160 days in specific conditions [25]. Less is known about the lifespan of *D. suzukii* in field conditions; flies were shown to live between 21 and 66 days in cherry orchards during the months of May–August in Japan [26]. To survive low temperatures during winter months in some parts of the world, *D. suzukii* undergoes reproductive diapause, transitioning into a winter morph phenotype. This transition is mainly triggered by temperature [27], with resulting adults being larger and darker in appearance [28] and more tolerant to cold conditions [29]. Prevalence of the winter morph phenotype increases in the latter stages of the growing season, reaching ~99% in December in the Netherlands [30]. This morphological transition is also believed to be accompanied by a dispersal into semi-wild habitats, such as hedgerows and woodlands, where flies may benefit from sheltering in microclimates such as leaf litter to aid their avoidance of extreme temperatures [31,32]. At the start of the subsequent fruit growing season, overwintering females enter crops to lay eggs in host fruit [30].

The aim of this study was to investigate if and how SIT could be employed to suppress *D. suzukii* in a commercial setting where physical barriers did not prevent *D. suzukii* incursion. This was tested through regular and dynamically targeted releases of sterile male *D. suzukii* into a commercial strawberry crop grown in open polytunnels and the surrounding habitat. The site had a previous history of *D. suzukii* infestation and sterile male *D. suzukii* were released from before the onset of wild *D. suzukii* activity in the spring of 2021 (week 15) through to the end of harvest (week 41) in the autumn of 2021.

## 2. Materials and Methods

### 2.1. Drosophila suzukii Rearing Conditions

The *D. suzukii* colony originated from wild flies collected at NIAB EMR (Kent, UK) from infested cherries in 2019. The culture was maintained in 30 × 30 × 30 cm cages with an average population size of approximately 4000–6000 *D. suzukii* per cage at NIAB EMR and, since October 2020, also at BigSis (Reading, UK). The rearing conditions were 16 L:8 D photoperiod, 24 °C (±2 °C), and 65% RH. *Drosophila suzukii* were maintained with a modified *D. suzukii* cornmeal–agar media containing 0.8% agar, 8% cornmeal (coarse organic polenta), 10% granulated sugar, 4% Bakers instant dry yeast (*Saccharomyces cerevisiae*) and 0.3% propionic acid.

### 2.2. Production of Sterile Male D. suzukii

For reasons of commercial protection, the production and sterilisation process of *D. suzukii* is not fully described in this paper, which focuses on the suppression of *D. suzukii*. However, methods to produce sterile *D. suzukii* are described elsewhere [21,22]. Males used for the trial were sterilised with X-rays, having been manually selected with fine paint brushes on fly pads under CO_2_ anaesthesia (Flystuff 59—122B Benchtop Flowbuddy Complete system; Genesee Scientific, San Diego, CA, USA) connected to water bubblers (Flystuff 59—180 CO_2_ bubbler on 500 mL flask with a No. 7 rubber stopper) to prevent males from dehydrating. The sterile males were marked with UV fluorescent powders (S-2800 Series Water-Based Acrylic Paint; Bioquip products, Inc., Compton, CA, USA; colours used—yellow, blue, red and orange) [33] to allow identification upon recapture. Sterile marked males were counted and groups of 100 were placed into vials (flat-bottom polypropylene tubes; 152 mm length x 26 mm diameter) containing ~5 mL of sugar–agar media (2% *w/v* agar, 5% *w/v* sucrose, 0.3% *v/v* acetic acid). Vials were kept at room temperature (~20 °C) until release, between three and seven days after sorting.

### 2.3. Sterility Validation Assay

To verify the sterility of the flies, irradiated and non-irradiated males were placed individually into vials containing ~5 mL of cornmeal–agar media, 40 vials in total, 20 with irradiated and 20 with non-irradiated males. To increase the probability of mating and the number of eggs collected in the vials, three virgin females were added to each vial containing a single male. Flies were allowed to recover from CO_2_ anaesthesia for 48 h and then transferred to new vials containing cornmeal–agar media dyed with red natural food colouring (The Pantry Red Food Colouring, 6 mL of dye per litre of diet) to facilitate the identification and counting of laid eggs. After 24 h, all flies were removed from the vials and the number of eggs was counted to obtain fecundity (number of eggs laid per female per 24 h). Vials with eggs were incubated for ten days at 24 °C (±2 °C), 16 L:8 D photoperiod and 65% RH and the number of eggs that hatched and became pupae was counted to calculate the fertility of the males (percentage of eggs that produced pupae).

A similar procedure was repeated over the course of the releases using flies randomly sampled from the release vials. At least 30 irradiated males were selected each time and these were distributed in groups of 10 per vial. A total of 10 virgin females were added to each vial. A table showing the number of eggs and pupae generated, average fecundity and average fertility of the sampled flies over the course of the trial is presented in Supplementary Table S1.

### 2.4. Study Sites and Releases

**Figure 1:**
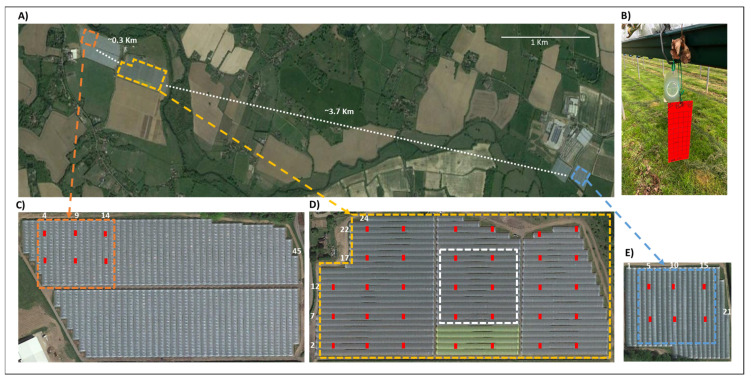
Relative position of experimental strawberry fields and monitoring traps within fields. (**A**) Satellite image of the region in Kent, UK, highlighting the position of the SIT-treated (dotted yellow lines) and untreated control sites (site 1—orange dotted lines; site 2—blue dotted lines). The distance between the sites is also shown. (**B**) Red sticky trap with a dry lure (Russell IPM) used to monitor the abundance of wild female SWD in the three sites. Their positioning is indicated by red rectangles on the images of individual sites. (**C**) Control site 1 and the position of six red sticky traps distributed among three tunnels. (**D**) SIT-treated site and the position of 33 traps distributed amongst 16 tunnels. (**E**) Control site 2 and the position of six red sticky traps distributed among three tunnels. Traps were distanced equally in the 3 sites, ~50 m within rows of polytunnels and 5 polytunnels apart (~40 m) from each other. The white rectangle in (**D**) represents a 1.4 ha “core area”, which was reproduced in each of the control sites. Photos were extracted from Google Earth.

The study was conducted at three commercial strawberry (*Fragaria × ananassa*) field sites in Kent, UK (Figure 1). The control sites were selected amongst available comparable crop fields based on historical data provided by the company’s *D. suzukii* monitoring programme. All three sites historically had relatively similar *D. suzukii* infestation levels based on available data of weekly catches of 1–2 liquid-baited traps during the 3 years prior to the trial (2018–2020), apart from untreated control site 1, for which data was available for 2019 and 2020 only. These data do not provide a robust comparison between the sites but do confirm, especially compared to other sites considered, a similar infestation characteristic. Supplementary Figure S1 presents the mean number of total *D. suzukii* (males plus females) captured in those traps in previous seasons. The surrounding landscape at the three sites consisted of strawberry fields, arable land, and hedgerows including *Rubus* spp. The different profiles of each site may have affected the dynamics of invasion from overwintering refugia; however, the historic data suggests that the net effect was broadly comparable.

The treated site was located approximately 0.3 km from control site 1 and 3.7 km from control site 2 (Figure 1A). All three sites were owned by the same company and received similar management, including a tabletop growing system (~1.3 m above the ground), open-ended polytunnels, biological control agents, beehives to increase pollination and chemical pesticide sprays against insect pests and diseases. Supplementary Table S2 shows the application dates, product name, active ingredient and application rates of all chemical insecticides used in the three sites during the course of the trial. The floor of the polytunnels was not treated with any plastic covering and was dominated by a cover of grasses. Strawberry plants (cv Zara; everbearing) were planted in coir grow bags in April 2021 and removed at the end of the season (October). Each polytunnel included 5 or 6 rows of tabletops with coir grow bags, making a total of ~52,000 plants per ha. The size of the SIT-treated site was 7.5 ha and it contained 70 tunnels of variable lengths. These were distributed in 22 rows of 3 blocks of tunnels each and 2 rows of 2 blocks of tunnels (24 rows in total). The untreated control site 1 was a 1.4 ha area chosen within a field containing 85 tunnels arranged in 2 blocks, 1 with 45 and another with 40 tunnels (45 rows in total). The untreated control site 2 was a 1.4 ha area within a field containing 21 tunnels in a single block.

The treated site received sterile male *D. suzukii* biweekly releases (SIT-treated site), whist the other two sites (untreated control sites 1 and 2) were similarly managed for the control of pests and diseases but without sterile male *D. suzukii* releases. Sterile male releases at the treated site began in April (week 15) and continued until October (week 41), when harvest ended. Two releases were made per week. Each Tuesday, flies were released in tunnels in odd-numbered rows; on Fridays, flies were released in tunnels in even-numbered rows. Sterile males were distributed evenly within the tunnels. Sterile flies were also released in the perimeter (surrounding hedgerows <20 m from polytunnel) on both days. For each release, typically 30% of vials were reserved for release in the perimeter (Supplementary Table S3). The distance between release points varied from 30 to 60 m depending on the number of flies being released. Sterile males were released manually from vials (100 males per vial), initially ~9000 males per week (in mid-April), rising progressively to around 60,000 males per week in October. Typically, 20% of the sterile flies released in a given day were targeted to areas where trapping had identified higher infestations of wild *D. suzukii* in previous days.

### 2.5. Population Monitoring

The main metric of the abundance of wild *D. suzukii* in the SIT-treated and control sites was the number of wild females captured per week on red sticky traps (red impact sticky board, size 10 × 24.5 cm—Russell IPM, Deeside, UK), since females cause economic damage [5]. Each red sticky trap (hereafter referred to as sticky trap) was supplemented with an attached semiochemical lure (SWD dry lure—Russell IPM) to increase the attraction of *D. suzukii* [34]. Sticky traps and their lures were placed approximately one metre above the ground, hanging underneath the tabletops (Figure 1B). Large foldback clips were used to give more weight and keep traps stable during windy days. The treated site had 33 sticky traps (Figure 1D), whilst each control site had 6 (Figure 1C,E). Sticky traps were evenly distributed and distanced ~50 m apart within tunnels and ~40 m apart between tunnels at all three sites. The two traps in the yellow tunnels at the SIT-treated site (Figure 1D) were not included in the analysis as these coloured polytunnel covers had been installed as part of a previous trial testing whether this could reduce *D. suzukii* infestation [35].

Red sticky traps with lures tend to catch fewer *D. suzukii* per trap per week than liquid-baited traps [36], but were preferred for this study because the drowning liquid bait could potentially wash off the fluorescent powder from recaptured sterile males. Thus, the weekly mean number of *D. suzukii* captured in bucket traps in previous seasons (2018–2020) (Supplementary Figure S1) are not directly comparable to the number of wild flies captured on red sticky traps in 2021.

Consideration was given to the possibility that the *D. suzukii* captured by the monitoring traps could make a significant contribution to the results. The experiment therefore used a similar density of trapping in the treated site (4.6 traps/ha) and each control sites (4.3 traps/ha). Furthermore, previous studies have investigated mass trapping as a possible method to control *D. suzukii* and found very low–no efficacy in controlling infestation levels in blueberry crops, both when traps were placed 5 m apart in the perimeter area [37] and 1.8 m apart within the crop area [38]. Thus, our trap design, where traps were spaced at least 40 m apart from each other, is very unlikely to trap sufficiently high numbers of *D. suzukii* to significantly impact local populations.

Sticky traps were collected (wrapped in clear side- and bottom-opening A4 punch pockets) and replaced twice a week at the treated site and once a week at the control sites from week 15 to week 41. However, traps were initially collected daily at the treated site to facilitate measurement of the longevity of released *D. suzukii* (see longevity experiment below). Lures remained in situ; a new second lure was added to each sticky trap after three months. After collection, all sticky traps were brought to the laboratory and numbers of marked (irradiated) males, wild males, and wild females were scored in a dark room using a light microscope and a UV torch (Eletorot ultraviolet torch 395 nm LED black light).

### 2.6. Longevity Experiment

To estimate the longevity of sterile male *D. suzukii* in the field, at the beginning of the suppression trial, sterile males released in the treated field were marked with intercalating fluorescent coloured powders and traps were replaced daily. No additional trap points were used for these experiments, only the ones already described above; furthermore, the lures were not changed, so the total number of trapped flies per week would not be affected. Four colours of fluorescent powder were rotated (yellow, blue, red and orange) to mark the flies and identify the release date of each cohort. Daily replacement of traps allowed us to track the survival of each cohort. Following the same release protocol described in 2.4, groups of approximately 5000 flies were released each time. The experiment was repeated five times using different colours (one colour was repeated 14 days after the first release), but two of the repeats did not result in a significant fit for regression models and were not used for further calculations.

### 2.7. Induced Field Sterility of Wild Female D. suzukii

To estimate the induced field wild female *D. suzukii* sterility caused by the release of the sterile males in the treated field, live females were captured from week 30 onwards. Suzukii Traps (Suzukii Trap; red base/transparent top—Russell IPM) were deployed at the treated and control sites. These were intended to attract, but not kill *D. suzukii*. In total, 19 Suzukii Traps were progressively deployed and assessed twice per week, 15 were set up at the treated site (six at the perimeter and nine under the polytunnels), whilst two were set up at each of the control sites (one at the perimeter and one under the polytunnels). Up to week 36, each Suzukii Trap was baited with a dry lure (as described above), ~20 blueberries, ~20 g frozen raspberries (included in a flat-bottom 70 mL yellow-lidded polypropylene container), and ~2 g Bakers yeast (sprinkled on blueberries) (Supplementary Figure S2). From week 37 onwards, the bait was switched to cotton wool soaked with ~115 mL liquid gasser (Gasser SWD Attractant-RIGA AG, Ellikon a. d. Thur, Switzerland), which proved to be more efficient in attracting and trapping wild female *D. suzukii*. In addition, each Suzukii Trap (irrespectively of the type of bait) contained a vial with ~10 mL of sugar–agar media (2% *w/v* agar, 5% *w/v* sucrose, 0.3% *v/v* acetic acid) as a source of food. On each assessment, Suzukii Traps were collected and replaced, and returned to the laboratory. Cotton wool and masking tape were used to seal the exit holes and prevent *D. suzukii* from escaping during transport. In the laboratory, all dipterans were collected from the Suzukii Traps using a laboratory pump (Dymax 14; Charles Austen Pumps Ltd, Byfleet, UK.) and/or anesthetized (placed in freezer for up to 3 min and 30 s). Then, female *D. suzukii* were selected using CO_2_ and placed—individually up to the fifth female and combined in groups of five thereafter—in vials with cornmeal media coloured with a food red dye (The Pantry Red Food Colouring, 6 mL per litre of diet). Females were left for 48 h to recover from the CO_2_, and then moved to a new vial to lay eggs for 24 h. When females did not lay eggs during this period, they were left in the vial for other 24 h. Next, females were removed, the number of eggs counted, and fecundity calculated. Ten days later, the number of pupae was counted, and fertility determined (as above). To account for a sudden drop in the number of eggs laid by the captured females, from early October onwards, the recovery period before egg laying was increased up to a maximum of 15 days. We speculated that this drop in fecundity was caused by cooler weather conditions recorded (data not shown).

### 2.8. Data Analysis

#### 2.8.1. Sterility Validation Assay

Mean fecundity and fertility for the sterility validation assay were calculated for each group of females that mated with either non-irradiated or irradiated males. Data were analysed with the software R (version 4.1.1) [39]. Females that did not lay eggs were removed from fertility analysis. Data were tested for normality using the Shapiro–Wilk tests. One-way ANOVA tests were used for fecundity data, whilst Kruskal–Wallis tests were used for fertility data.

#### 2.8.2. Sterility of Released Male *D. suzukii*

Mean fecundity and fertility of females captured in the filed were also calculated and analysed with the software R (version 4.1.1) [39]. Females that did not lay eggs were removed from the fertility analysis. Data were tested for normality using the Shapiro–Wilk test. Kruskal–Wallis tests were used for both fecundity and fertility datasets. To facilitate the management of the data, we first compared fecundity and fertility at both control sites; upon finding no significant differences, we then combined control site data for further analysis.

#### 2.8.3. Longevity and Standing Crop Calculations

For each released cohort, the decline in daily recapture numbers over time was assumed to be due to mortality. Hence, the rate of decline was used to estimate longevity using MRR statistics adapted from Niebylski and Craig [40]. Removal of marked insects from the population due to recapture could confound the analysis as it would be erroneously interpreted as mortality. In many studies, the proportion of released flies recaptured was small enough that adjustment for recaptured insects was not implemented. However, due to the high proportion of recaptures in these experiments (~10%), the number of *D. suzukii* of each colour recaptured during the daily trapping was adjusted to account for the number of *D. suzukii* recaptured on the previous days. This was carried out by multiplying each value by the original release number and dividing by the number of uncaptured *D. suzukii*. This effectively estimated and added back the number of previously captured *D. suzukii* that would have been captured had they not been removed from the experiment. Next, the three-day moving averages of the adjusted recaptures were calculated and transformed to Log_10_. The slope of the regression line of Log_10_ (adjusted recaptures) versus day of trapping was used to calculate the probability of daily survival (PDS), using

PDS = 10^slope^

Both half-life (HL) and average life expectancy (ALE) were derived from the PDS, where

HL = Log_e_(0.5)/Log_e_(PDS)

and

ALE = −1/Log_e_(PDS).

Regression lines were fitted using GraphPad Prism (Graph-Pad Software, San Diego, CA, USA); this identified that three of the five replicates had a significant fit. The summary statistics, best fit values, 95% confidence intervals, goodness of fit and normality tests for these three replicates were calculated (Supplementary Table S4). Final PDS, HL and ALE, and the corresponding standard deviations, were derived from the mean of the three replicates.

The daily estimated number of sterile males predicted alive at any given point (estimated standing crop) (SS) was calculated from the number of sterile males released (R) and the estimated PDS using the following equation:

SS_dx_ = (SS_dx−1_ × PDS) + R_dx_,

where the number of alive sterile males in each day (S_dx_) equals the number of sterile males alive the day before (S_dx−1_) multiplied by the PDS plus the number of sterile males released on a given day (R_dx_). The standing crop of wild males (SW) was calculated based on the weekly average standing crop of sterile males ($\bar{\mathrm{SS}}$) and the ratio of sterile (S) and wild (W) males trapped during that week. The following equation was used to calculate the weekly standing crop of wild males:

$$\mathrm{SW}=W/S\times \bar{\mathrm{SS}}$$

#### 2.8.4. Suppression of Wild *D. suzukii* Population

The trapping interval varied between sites (biweekly for treated site and weekly for control sites) and over time (daily for the longevity MRR study). Therefore, the standardised unit of measurement analysed was the total number of females per sticky trap per week. The effect of SIT treatment on the number of females captured over time was analysed with a Poisson GLM mixed model using the lme4 1.1–27.1 package [41] in R (version 4.1.1) [39]. The number of flies captured on sticky traps per week were treated as repeated measurements and, as we had only one treated field, polytunnels were considered as replication units, set up as crossed factors. The random effects were polytunnel—trap week (pseudo replicates); polytunnel—traps (repeated measures). Treatment effects were measured with a likelihood ratio test followed by post hoc test using the emmeans 1.7.0 package [42].

## 3. Results

### 3.1. Sterility Validation Assay

**Figure 2:**
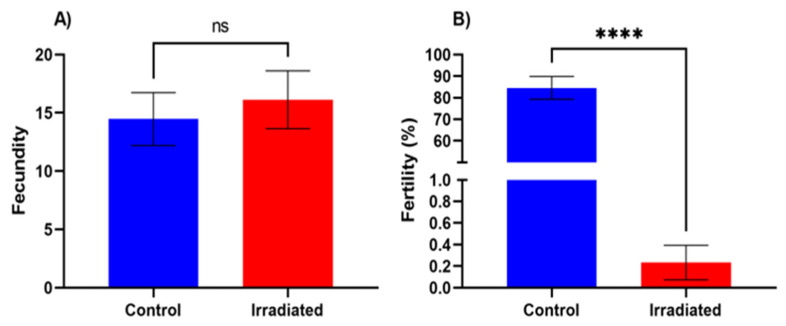
(**A**) Fecundity of the females (±SE) that mated with control (non-irradiated) and irradiated males. Fecundity is presented as the mean number of eggs laid per female in a 24 h period. (**B**) Fertility (%) (±SE) of females that mated with control (non-irradiated) and irradiated males. Fertility is presented as the percentage of eggs that hatched and developed to pupa stage. ns—no significant difference between treatments; asterisks represent a significant difference between treatments (**** = *p* < 0.001; Kruskal–Wallis test).

The fecundity (numbers of eggs laid per female per 24 h period) of females that mated with non-irradiated males was 14.48 (±2.27 SE) eggs. This was not significantly different compared to the fecundity of females that mated with irradiated males (one-way ANOVA: F(1,38) = 0.24, *p* = 0.63), which laid 16.13 (±2.48) eggs (Figure 2A).

There was a significant reduction, >99.7%, of fertility (% egg–pupa) of females that mated with irradiated males (0.23%, ±0.16) relative to females that mated with non-irradiated (controls) 84.60% (±5.27) (Kruskal–Wallis: H = 24.84, df = 1, *p* < 0.001) (Figure 2B).

### 3.2. Sterile Male Releases

A total of 950,739 sterile male *D. suzukii* were released at the treated site throughout the trial (April–October), of which 619,604 were released under the polytunnels and 331,135 around the perimeter of the polytunnels. In total, 14,910 sterile male *D. suzukii* were recaptured on the traps placed under the polytunnels. This represents 2.41% of the flies released under the polytunnels. However, recapture rate fell progressively during the season from over 10% in April to less than 1% in October.

### 3.3. Longevity and Estimated Standing Crop

Across the three releases used to estimate longevity in field conditions, a total of 1503 *D. suzukii* were recaptured, representing 10.13% of the total number released. Based on the mean of the three releases, the PDS (±SD), HL (±SD) and ALE (±SD) of the sterile *D. suzukii* were 0.80 (±0.01), 3.19 (±0.18) days and 4.60 (±0.25) days, respectively (Table 1). Figure S3 presents the regression lines of the Log_10_ of the adjusted recapture rates of sterile *D. suzukii* over the 14-day trapping period for the 3 releases (replicates 1–3).

**Figure 3:**
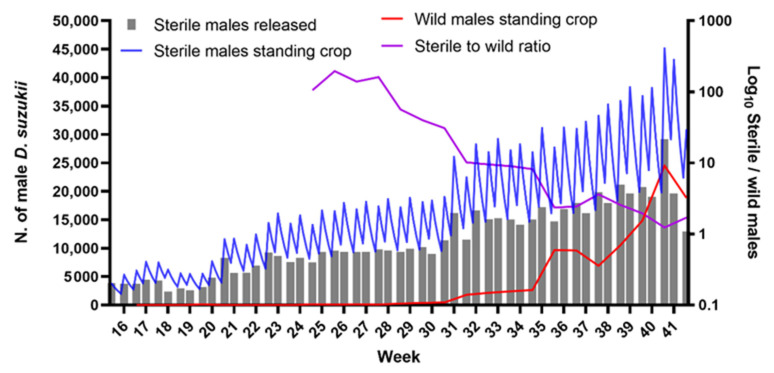
Number of sterile male *D. suzukii* released (grey), estimated standing crop of sterile (blue line) and wild (red line) male *D. suzukii* (left *y*-axis) and the ratio of sterile to wild captures (purple line, right *Y*-axis) on red sticky traps (red impact sticky board; 10 × 24.5 cm—Russell IPM), each with an attached dry lure (SWD dry lure—Russell IPM) at the SIT-treated site under polytunnels throughout the trial (April–October 2021). Sterile to wild male ratio before week 25 was excluded because no wild males were caught. The *y*-axis on the right represents the sterile to wild ratio on a log_10_ scale.

The standing crop of sterile males at the treated site varied due to the mortality of the flies and biweekly releases (Figure 3). Reflecting production capacity, it steadily increased throughout the season, from approximately 9000 sterile males per week in week 16 to approximately 60,000 in week 41. Approximately two-thirds of those males were released in the crop (Figure 3), the remaining were released in perimeter habitat. The estimated number of wild males (standing crop of wild males), which was calculated based on the ratio of sterile to wild males captured on the sticky traps placed under the polytunnels and the estimated standing crop of sterile males (as described above), was in the hundreds up to week 30. In week 31, the number of wild males was estimated at 1783 under the polytunnels. This rose to 18,817 by the end of the trial (week 41).

### 3.4. Field Sterility of Wild Female D. suzukii

**Figure 4:**
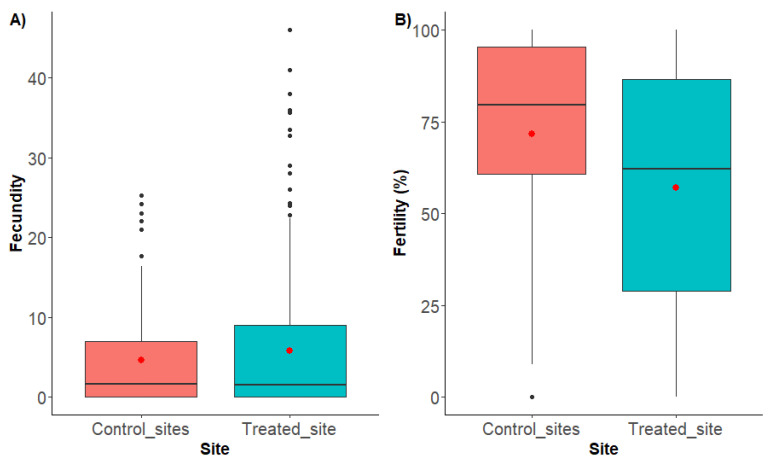
(**A**) Fecundity and (**B**) fertility of wild female *D. suzukii* collected from SIT-treated and control sites (combined) from week 30 to week 41. Fecundity is calculated as the number of eggs laid per female per 24 h. Fertility is calculated as the percentage of eggs that developed to pupae. Box plots represent the interquartile range of the median (horizontal line). Error bars represent variation of the data outside the interquartile range. Red dots represent the mean and black dots represent outliers. n = 1804 total females: 420 at untreated control sites; 1384 at SIT-treated site.

The fecundity of female *D. suzukii* did not differ significantly between control and treated sites (Kruskal–Wallis: H = 0.21; df = 1, *p* = 0.65). The mean fecundity of females at control sites was 4.62 (±0.73 SE) eggs per female per 24 h, similar to the mean from females collected at the treated site 5.79 (±0.57 SE) (Figure 4A). In contrast, the fertility of females collected at the control sites was 71.60% (±4.12% SE), which was significantly greater compared to the treated site 57.00% (±2.71% SE) (Kruskal–Wallis: H = 6.64; df = 1; *p* = 0.01) (Figure 4B). The number of wild females captured per week in the treated and control sites as well as the number of eggs laid by those females, the number of pupae generated from those eggs and the overall fecundity and fertility of the females are presented in Supplementary Table S5.

### 3.5. Suppression of Wild D. suzukii Population

The main metric used to measure wild female *D. suzukii* prevalence and hence estimate suppression was the mean number of wild female *D. suzukii* per sticky trap at each site over time. During the whole trial, a total of 520 wild females were recorded at the treated site, which is an average of 16.8 females per trap. At control sites 1 and 2, the average number of wild females per trap was 55.0 and 80.1, respectively. There was a significant effect of treatment (release of sterile male *D. suzukii*), and time (females captured throughout the season), including a significant effect on the interaction between both response variables (Table 2). This indicated that female captures were inconsistent throughout the season but also differed between sites.

**Figure 5:**
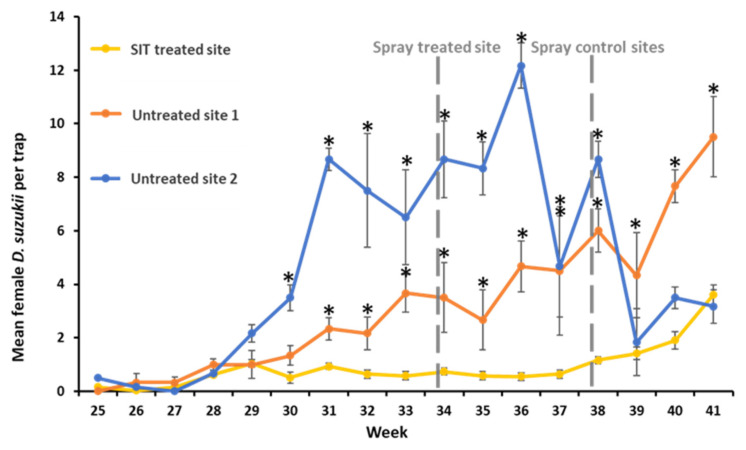
Mean numbers of female *D. suzukii* (±SE) captured per trap and per week at the SIT-treated and untreated control sites throughout the trial. Statistically significant differences (*p* ≤ 0.05; GLM mixed model) between the SIT-treated site (yellow line, n = 31) and each of the untreated control sites 1 (blue line, n = 6) and 2 (orange line, n = 6) are represented by an asterisk above the error bars for each week. Foliar insecticide application (cyantraniliprole (100 g L^−1^) at a rate of 750 mL ha^−1^) at the SIT-treated and untreated control sites are highlighted by vertical grey dashed lines.

The treated site had a sustained lower number of wild female *D. suzukii* over the season compared with the control sites (Figure 5). Up to week 27, the number of females per trap was similar and close to zero at all three sites. After weeks 29–30, the mean number of females per trap started to increase exponentially at both control sites but remained relatively flat up to week 37 at the treated site. The increase in wild female *D. suzukii* in the control sites followed the appearance of the first fruit. Fruit ripening started in mid-June (weeks 24–26) and cropping stopped in the end of October (week 42). As part of the farm’s IPM programme, an insecticide spray took place at all three sites; however, it occurred at different times during the trial. The treated site was sprayed with cyantraniliprole (100 g L^−1^) at a rate of 750 mL ha^−1^ during week 34, whilst both control sites received the same treatment four weeks later (week 38) (Figure 5; Supplementary Table S2).

**Figure 6:**
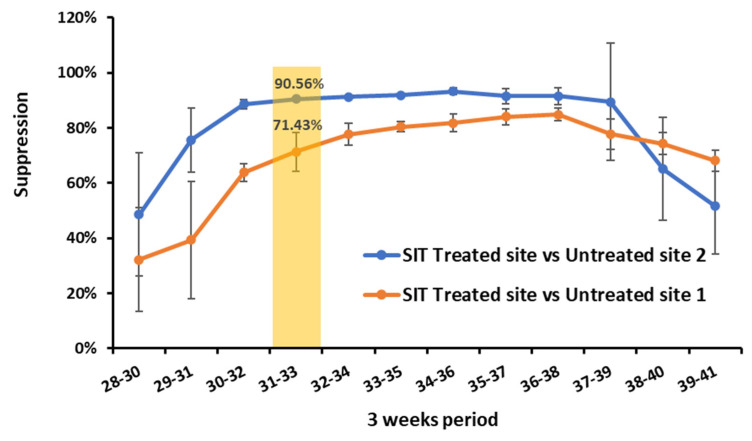
Suppression (%) of wild female *D. suzukii* population at the SIT-treated site compared to the two untreated control sites. Suppression is derived from the three-week moving averages of female *D. suzukii* per red sticky trap, comparing the SIT-treated site and the untreated control sites (suppression = 1- (treated site/control site)). The solid yellow box highlights the highest suppression rate achieved at the SIT-treated site in comparison to control sites prior to the insecticide spray at the SIT-treated site. Error bars represent the SE of the three-week value.

Due to the volatility of weekly count data, suppression was calculated based on a moving three-week average basis (Figure 6). The greatest suppression prior to week 34 was in weeks 31–33, when there was a pest population suppression of 71.43% and 90.56% at the treated site in comparison with control sites 1 and 2, respectively.

## 4. Discussion

During more than 60 years of operational use, SIT has proven to be a very effective and environmentally safe method of insect pest control. It has been used against important pests in agriculture, public health, animal welfare and other sectors. For an in-depth review of SIT, see [10].

In the present study, we demonstrated, for the first time, the feasibility of using SIT to control *D. suzukii*, a major global economic pest of soft and stone fruit, in a commercial setting without effective physical barriers, i.e., open-ended polytunnels using coco–coir substrate bags on tables. We provided evidence suggesting that an early SIT intervention, beginning from the spring as *D. suzukii* emerge from overwintering, comprising regular releases of sterile males, can suppress the reported exponential growth of *D. suzukii* [43] on everbearing strawberries cultivated in this setting. By starting the releases of sterile males early in the season, when the wild populations were still low, and maintaining a substantial sterile population throughout the season, we were able to sustain a low level of infestation at the treated site (Figure 5).

We were able to attribute the suppression due solely to SIT only up to and including week 33 due to the application of chemical insecticide at the treated site (Figure 6). Despite this, in comparison with the two untreated control sites, suppression ratios of up to 71% and 91% were achieved by SIT treatment alone, measured on a three-week moving average. However, since the mean number of wild females per trap differed substantially between control sites over the course of the trial, it is possible that the suppression attributed to the SIT treatment might have been understated or overstated due to natural variations. It is also possible that environmental factors were less favourable for the population growth of *D. suzukii* in the tested region in 2021 compared with previous years. This could have contributed to the success of this trial by reducing the denominator of the sterile to wild ratio. Similarly, strawberry fields do not typically experience as high *D. suzukii* infestations as some other crops, such as blackberries. Hence, trials in seasons with higher *D. suzukii* pressure and in other crops will provide further insight regarding the efficacy of SIT to sustain control of *D. suzukii* in these circumstances.

We also showed that when the control sites were sprayed with chemical insecticide, a substantial or sustained suppression of *D. suzukii* infestation compared to the treated site was not achieved. Interestingly, the insecticide application did not substantially reduce numbers of female *D. suzukii* in untreated control site 2 and there was no detectable effect on the SIT-treated site. Finally, we demonstrated that SIT has the potential to be applied successfully on individual commercial farms to control the local wild *D. suzukii* population. Given that *D. suzukii* is one of the most economically important pests of soft and stone fruit in the world, a method of control that does not rely on broad-spectrum insecticides is urgently needed. SIT, an environmentally friendly pest-management tool, is especially well suited for continuous harvest crops, as it does not require preharvest intervals. Additionally, when SIT is used preventively, it does not rely on an infestation threshold, which usually is already associated with some degree of crop damage and economic losses to growers.

While *D. suzukii* suppression was demonstrated in only one season, the sustained weekly suppression of *D. suzukii* trap catches provide evidence of *D. suzukii* control in the crop. This is a comparable approach to other studies assessing the feasibility of using SIT against other species with multiple generations per season [44,45].

The recapture rate of released sterile male *D. suzukii*, which were all marked, in the treated area was typically ~1% in late summer, when *D. suzukii* infestation was highest, further validating the hypothesis when the trap layout was designed that our monitoring traps did not contribute to *D. suzukii* suppression in our study sites. The trap density used to monitor the SIT-treated site was 4.6 traps/ha. A recent study looking at the potential of mass trapping to suppress *D. suzukii* populations in sour cherries showed that, depending on the attractant and the environment, the required trap density to produce a grid of traps with contiguous attraction radii varied from 25 to 300 traps/ha [46].

Sexual sterility can be induced by different means; these include chemical agents, genetic manipulation, and ionising radiation (X-rays and gamma rays). The main advantage of ionising radiation, in particular X-rays, is that it does not create or make use of potential environmental contaminants such as radioactive waste, chemosterilants or transgenic material. *Drosophila suzukii* is relatively resilient to radiation-induced somatic damage and the fitness penalties associated with it [47]. In a first attempt to identify an optimal SIT radiation dose for *D. suzukii*, Lanouette et al. [22] exposed four-day-old pupae to increasing doses of gamma radiation (0, 30, 50, 70, 80, 90, 100, 120 Gy) and measured the effect of these doses on adult emergence, longevity, fecundity and fertility of the survivors. The authors showed that none of the radiation doses had a significant effect on the parameters tested, apart from fertility and fecundity. Even with the highest dose (120 Gy), which reduced fertility by more than 99%, no apparent impact on *D. suzukii* fitness was observed. A follow-on study by the same authors concluded that the irradiation dose of 120 Gy applied to four-day old pupae did not affect male mating performance under both non-competitive and competitive conditions in a laboratory setting [24]. Previous studies using gamma rays reported that sterilization doses of up to 200 Gy on pupae had no effect on the adult emergence, flight ability, sex ratio, longevity or reproductive behaviour of *D. suzukii* [30,33]. Similar results were reported by Sassù et al. [48] investigating the effects of irradiating *D. suzukii* pupae under low and normal oxygen conditions. The authors reported that radiation doses up to 220 Gy under both atmosphere treatments had no effect on emergence rate and flight ability of *D. suzukii*. Altogether, these studies suggest that *D. suzukii* can withstand radiation doses required to induce high sterility levels without major compromise to fitness.

Our study is the first to investigate the use of X-rays to sterilise *D. suzukii* for SIT. Our results corroborated those from the literature using gamma rays and demonstrated that sterile *D. suzukii* were able to survive in field conditions. The calculated PDS of 0.8 indicated a daily survival rate that translates to an average life expectancy (ALE) of 4.6 days in field conditions. Sterile mass-reared males of *Anastrepha ludens* (Loew) and *A. obliqua* (Macquart) released on a mango orchard presented an ALE of 2.2 and 4.2 days, respectively, and these were not statically significant from wild males of the same species, which presented ALE of 3.1 and 4.3 days, respectively [49]. We were not able to estimate the life expectancy of wild *D. suzukii* during this trial so we cannot infer whether sterile males lived for a shorter period than wild males in field conditions. Furthermore, the measurement was taken at the start of the season when it was relatively cold and the crop foliage was less developed, so it is possible that flies released later in the season would have presented a different lifespan.

The discrepancy between the fertility levels of females mated with irradiated males in the lab with that of females captured from the SIT-treated field could be attributed to two factors. First, the fertility of the females captured in the SIT-treated field are presented as the overall fertility of all females captured during this experiment (week 30 to 41). It is likely that, whilst some of these females mated with irradiated males, others mated with wild fertile counterparts, including in adjacent areas, before invading the treated site. Second, some of the females might have mated with both wild and irradiated males as *D. suzukii* is known to remate [23,24,50]. The order in which females mate with sterile or fertile males seems to influence the outcome, with the first males having a greater influence on the fertility of the eggs [50]. Both factors could then be influenced by the sterile to wild ratio achieved in the SIT-treated site.

The decision to release only sterile males assumed that sterile females would interfere with the sterile males’ success in mating with wild females. In a study comparing single-sex (male only) versus bi-sex (male and female) releases of sterile *C. capitata* in coffee plantations in Guatemala over three years, it was concluded the male-only releases increased by three–fivefold the levels of induced sterility of eggs laid by wild females [51]. By not releasing sterile females, we also removed the potential for damage to the crop, even though such females are fully sterile and typically will not lay eggs [22].

One of the main misconceptions of SIT is that it must be applied to a large area for it to be effective. Whilst this might be true if eradication is the final goal, it is well established that SIT can suppress pest populations to below economic thresholds at much smaller scales. This is very relevant to commercial models that rely on selling insect control to growers for individual farms. The smallest practical field size that can be treated is inversely related to the dispersal of the pest. The daily dispersal of *D. suzukii* has been estimated to be 28.8 m in highbush blueberries, with a maximum dispersive distance for 95% of the population estimated at ~90 m [52]. In cherry and citrus orchards, the daily dispersals have been estimated to be 90 m and 200–300 m, respectively [53,54]. In a study looking at long-distance dispersal at a regional scale, *D. suzukii* was shown to be able to disperse up to 9000 m, migrating to higher altitudes in spring and summer and lower altitudes in autumn and winter [55]. This long-distance dispersal was believed to be facilitated by seasonal breezes induced by differences in temperature between area elevations. In this study, we saw a very low number of sterile males recaptured in the untreated control site 1 (8 out of a total of 925,042 sterile males released), which was 300 m away from the SIT-treated site. This, together with the estimated life expectancy, suggest that the flies do not disperse long distances in the studied settings.

The results presented here indicate that SIT can be employed successfully to control *D. suzukii* at individual commercial farms where the crop is cultivated under open-ended polytunnels. Our infestation-responsive release approach distributed a baseline number of sterile insects throughout the field and added extra *D. suzukii* where increased numbers of wild insects were detected. These targeted releases were repeated twice a week throughout the season. In addition, we released typically one third of our sterile males at the field perimeter areas, thus adding a buffer zone that acted as first line of defence against invasion. *Drosophila suzukii* utilise field margins containing non-crop plants as refugia and these might become breeding sites if containing an alternate host, such as wild blackberries [56]. The possibility of targeting these areas is clearly a major advantage of SIT compared with chemical insecticides, which are not licensed to treat non-crop areas.

## Figures and Tables

**Table 1:** Slopes of the regression lines, probability of daily survival (PDS), half-life (HL) and average life expectancy (ALE) of sterile male *D. suzukii*.

Replicate	Slope	PDS	HL (Days)	ALE (Days)
**1**	−0.09	0.81	3.37	4.87
**2**	−0.10	0.80	3.15	4.55
**3**	−0.10	0.80	3.03	4.37
**Mean (±SD)**	−0.09 (±0.01)	0.80 (±0.01)	3.19 (±0.18)	4.60 (±0.25)

**Table 2:** Summary of test statistics for the likelihood ratio tests analysing the interaction between *D. suzukii* infestation, SIT treatment and time of the year (week).

Response Variable	Npar	Deviance	Chisq	Degrees of Freedom	Pr (>Chisq)
**Null**	4	2388.1			
**Week**	21	2215.1	173.06	17	<0.001
**SIT Treatment**	23	2161.8	53.28	2	<0.001
**Week × SIT Treatment**	53	2014.6	147.19	30	<0.001

## Data Availability

The datasets will be available from the corresponding author upon reasonable request.

## Supplementary Materials

The following supporting information can be downloaded at: https://www.mdpi.com/article/10.3390/insects13040328/s1, Figure S1: Mean number of *D. suzukii* captured per week in liquid-baited traps in the three seasons before the study (2018–2020), Figure S2: An example of a Suzukii Trap (Suzukii Trap; red base/transparent top—Russell IPM) used to collect wild females in the field. Figure S3: Regression lines of log_10_[adjusted recaptures] of released sterile male *D. suzukii* conducted as part of the longevity experiment at the SIT-treated site, Table S1: Fertility of flies sampled from release vials, Table S2: Insecticide application records in the SIT-treated and control sites during the 2021 season, Table S3: Number and percentage of sterile males released under polytunnels and at the perimeter, Table S4: Summary statistics, best fit values, 95% confidence intervals, goodness of fit and normality tests of the three releases of sterile male *D. suzukii* conducted as part of the longevity experiment at the SIT-treated site, Table S5: Fertility of wild female *D. suzukii* collected from the control and SIT-treated sites from week 30 to week 41.
