# Supplementary material for: Field Suppression of Spotted Wing Drosophila (SWD) (Drosophila suzukii Matsumura) Using the Sterile Insect Technique (SIT)
Source: Insects. 2022 Mar 26;13(4):328. doi: 10.3390/insects13040328 (PMC9031279; doi:10.3390/insects13040328)
Supplement: Supplementary file 1 [file insects-13-00328-s001.zip › insects-1596504-supplementary.pdf]

Supporting Information

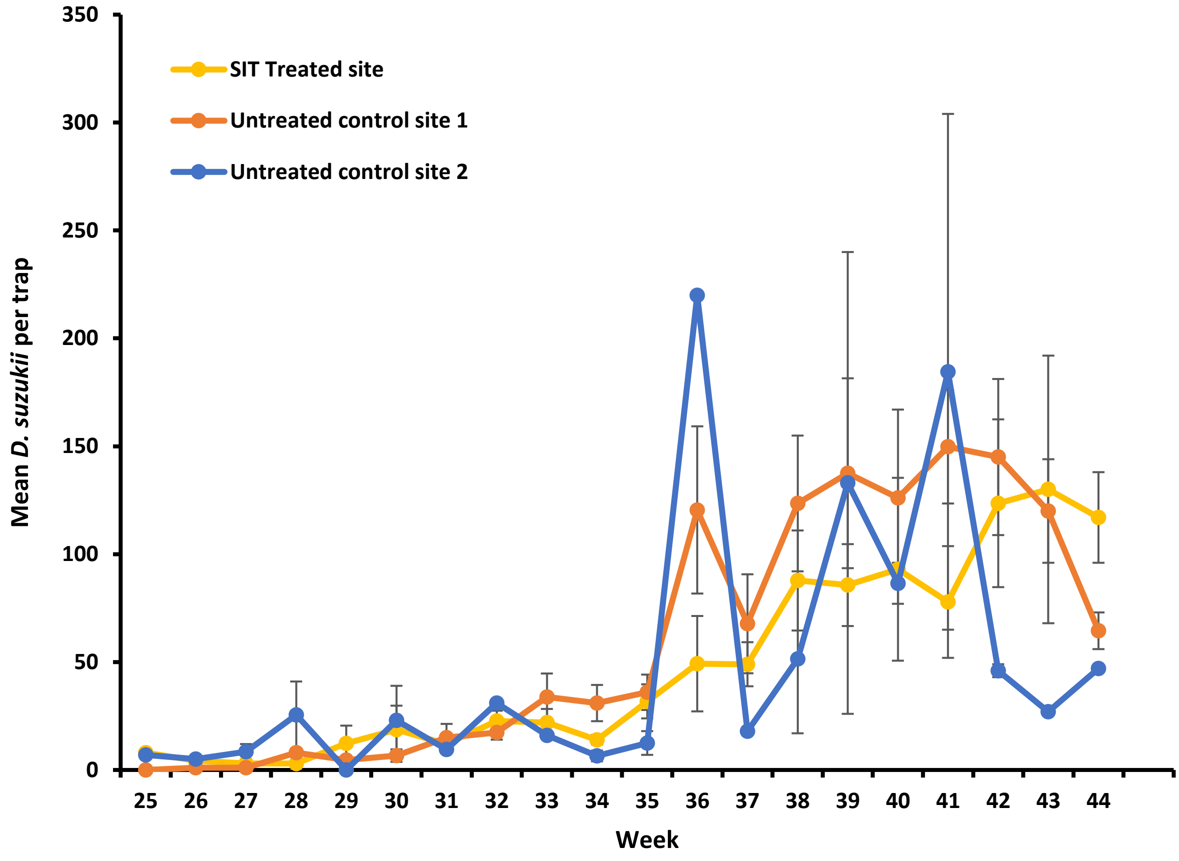

Figure S1. Mean number of *D. suzukii* captured per week in liquid baited traps in the three seasons before the study (2018-2020). Sites presented relatively similar levels of *D. suzukii* infestation in previous years. The type of liquid bait varied from season to season and the number of traps varied from site to site and season to season. For most of the data points presented, data were collected from at least two to three traps - one trap per site per year; a few data points had two. There were no data available for untreated site 1 in 2018. Error bars represent the standard error of the mean. Lack of error bars means data derived from a single trap.

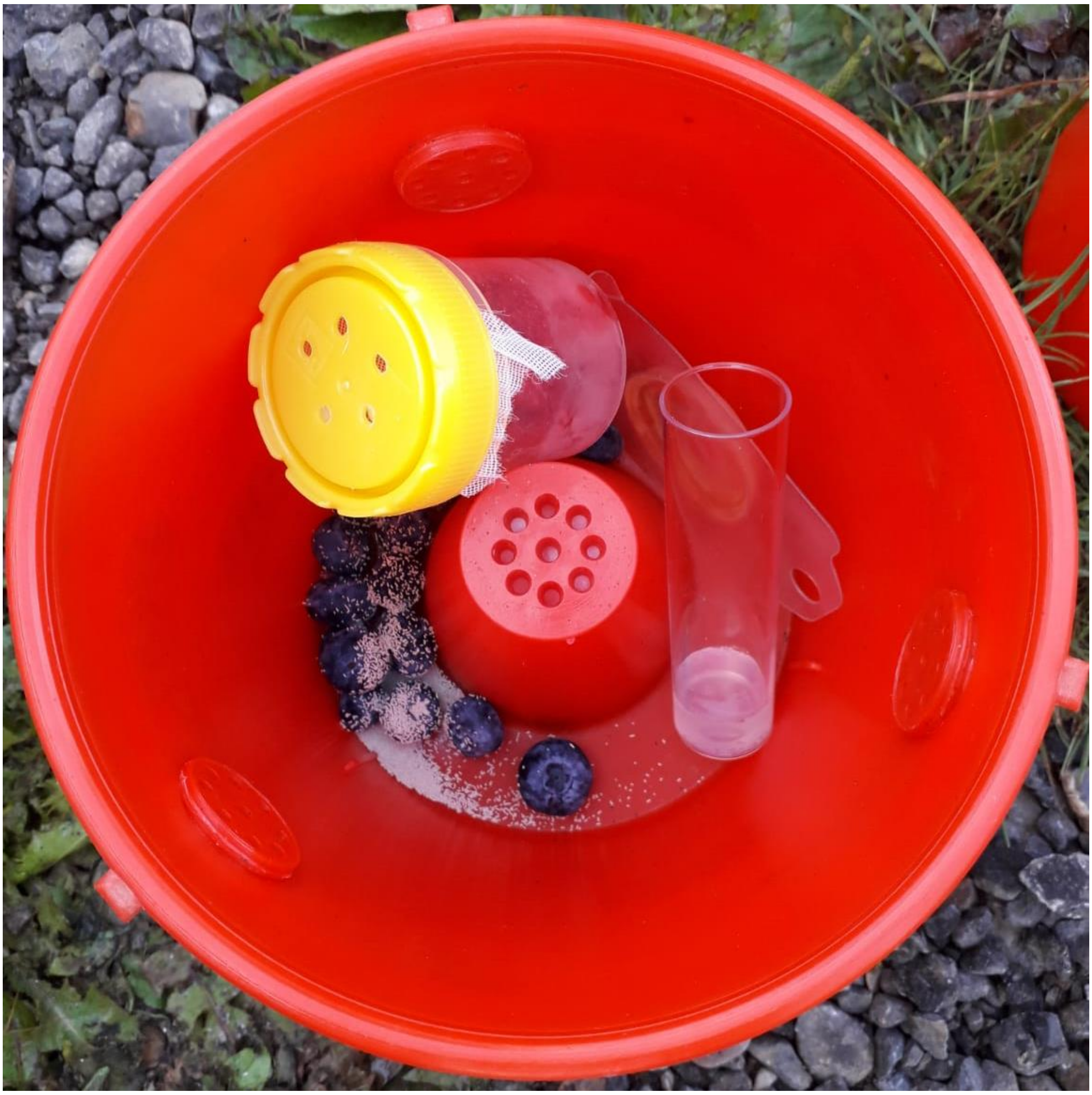

Figure S2. An example of a Suzukii Trap (Russell IPM Suzukii Trap; red base/transparent top) used to collect wild females in the field. Suzukii Traps up to week 36 included a dry lure (Russell IPM SWD dry lure), ~20 blueberries, ~20 g frozen raspberries (included in a flat-bottom polypropylene 70 ml yellow-lidded container), and ~2 g Bakers yeast (sprinkled on blueberries), to attract *D. suzukii*, and a vial with ~10 ml of sugar-agar media (2% w/v agar, 5% w/v sucrose, 0.3% v/v acetic acid) as a source of food. Holes were made on the yellow lids to maximize odour release of the raspberries but a mesh was placed under the lid to avoid *D. suzukii* from entering the container.

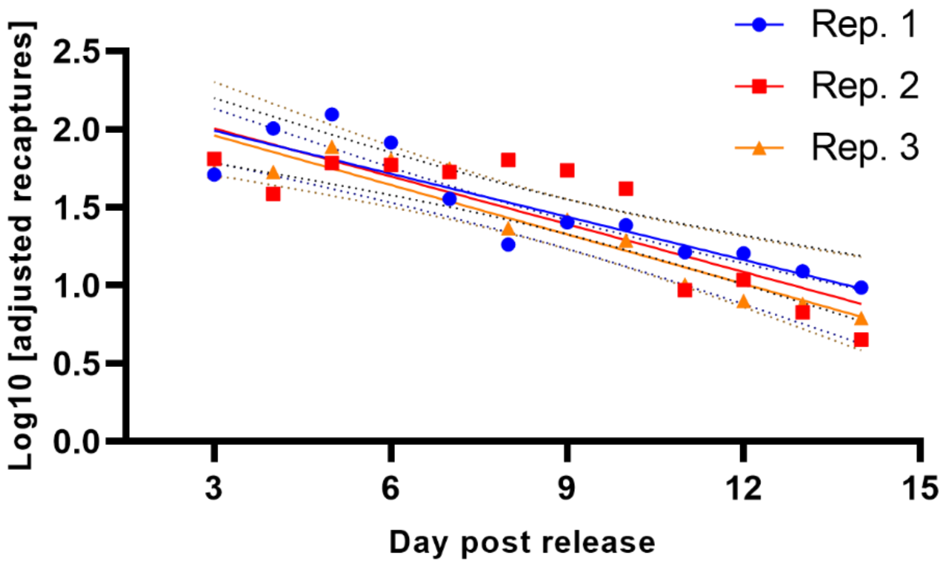

Figure S3. Regression lines of $\log_{10}$ [adjusted recaptures] of released sterile male *D. suzukii* conducted as part of the longevity experiment at the SIT treated site. Recaptures were adjusted to account for males removed from the trial (recaptured on previous days) and converted to three days moving averages. Doted lines represent the confidence bands of the regression lines. Rep. = replicate.

Table S1. Fertility of flies sampled from release vials. Table shows the number of irradiated males sampled from the vials each day, the number of virgin females used in the crosses, the number of eggs laid by the females in a 24 hours period and the number of pupae generated after 10-day incubation of the eggs. Fertility is expressed as percentage of eggs that produced pupae.

Irradiation date	No. Males sampled	No. Females	No. Eggs	No. Pupae	% Fertility
**16-Apr**	30	30	124	1	1.28
**20-Apr**	30	30	212	2	0.94
**23-Apr**	30	30	94	17	16.48
**04-May**	30	30	314	19	6.23
**07-May**	30	30	173	16	10.26
**18-May**	30	30	236	5	2.64
**25-May**	30	30	83	0	0.00
**28-May**	40	40	312	0	0.00
**01-Jun**	40	40	179	2	0.86
**08-Jun**	60	60	341	5	1.48
**22-Jun**	40	40	206	1	0.20
**02-Jul**	40	40	99	0	0.00
**13-Jul**	40	40	59	2	2.45
**20-Jul**	40	40	192	0	0.00
**23-Jul**	40	40	200	2	0.91
**06-Aug**	50	50	257	9	2.95
**10-Aug**	40	40	105	1	2.08
**13-Aug**	40	40	91	5	7.78
**17-Aug**	40	40	213	12	5.59
**20-Aug**	40	40	193	5	2.62

Table S2. Insecticide application records in the SIT treated and control sites during the 2021 season.

Site	Spray date	Week	Active ingredient	Application rate
**SIT treated site**	05-May	19	Spirotetramat (100g L^{-1})	1,000 mL ha^{-1}
	19-Aug	34	Cyantraniliprole (100g L^{-1})	750 mL ha^{-1}
**Untreated control site 1**	30-Apr	18	Spirotetramat (100g L^{-1})	1,000 mL ha^{-1}
	20-Sep	38	Cyantraniliprole (100g L^{-1})	750 mL ha^{-1}
**Untreated control site 2**	06-May	19	Spirotetramat (100g L^{-1})	1,000 mL ha^{-1}
	20-Sep	38	Cyantraniliprole (100g L^{-1})	750 mL ha^{-1}

Table S3: Number and percentage of sterile males released under polytunnels and at the perimeter.

			Number released			% of the total released	
**Date released**		**Week**	**Tunnels**	**Perimeter**	**Total**	**Tunnels**	**Perimeter**
**12/04/21**	Mon	15	3836	1020	4856	79.0	21.0
**16/04/21***	Fri	16	3704	800	4504	82.2	17.8
**20/04/21***	Tue	16	3777	1156	4933	76.6	23.4
**23/04/21***	Fri	17	4446	960	5406	82.2	17.8
**27/04/21***	Tue	17	4287	1105	5392	79.5	20.5
**30/04/21***	Fri	18	2334	3200	5534	42.2	57.8
**04/05/21**	Tue	18	2932	1904	4836	60.6	39.4
**07/05/21**	Fri	19	2572	2464	5036	51.1	48.9
**11/05/21**	Tue	19	3193	2160	5353	59.6	40.4
**14/05/21**	Fri	20	4814	1040	5854	82.2	17.8
**18/05/21**	Tue	20	8336	1870	10206	81.7	18.3
**21/05/21**	Fri	21	5635	3040	8675	65.0	35.0
**25/05/21**	Tue	21	5673	5250	10923	51.9	48.1
**28/05/21**	Fri	22	6900	5766	12666	54.5	45.5
**01/06/21**	Tue	22	9220	5600	14820	62.2	37.8
**04/06/21**	Fri	23	8586	5200	13786	62.3	37.7
**08/06/21**	Tue	23	7563	5600	13163	57.5	42.5
**11/06/21**	Fri	24	8303	8600	16903	49.1	50.9
**15/06/21**	Tue	24	7489	10200	17689	42.3	57.7
**18/06/21**	Fri	25	9312	8600	17912	52.0	48.0
**22/06/21**	Tue	25	9555	7300	16855	56.7	43.3
**25/06/21**	Fri	26	9345	7400	16745	55.8	44.2
**29/06/21**	Tue	26	9308	7400	16708	55.7	44.3
**02/07/21**	Fri	27	9387	7800	17187	54.6	45.4

**06/07/21**	Tue	27	9746	7200	16946	57.5	42.5
**09/07/21**	Fri	28	9600	7700	17300	55.5	44.5
**13/07/21**	Tue	28	9384	7200	16584	56.6	43.4
**16/07/21**	Fri	29	9911	7400	17311	57.3	42.7
**20/07/21**	Tue	29	10184	7200	17384	58.6	41.4
**23/07/21**	Fri	30	8938	8500	17438	51.3	48.7
**27/07/21**	Tue	30	11332	5200	16532	68.5	31.5
**30/07/21**	Fri	31	16202	5200	21402	75.7	24.3
**03/08/21**	Tue	31	11535	5200	16735	68.9	31.1
**06/08/21**	Fri	32	16589	5400	21989	75.4	24.6
**10/08/21**	Tue	32	15055	6500	21555	69.8	30.2
**13/08/21**	Fri	33	15232	6500	21732	70.1	29.9
**17/08/21**	Tue	33	15000	6500	21500	69.8	30.2
**20/08/21**	Fri	34	14107	7100	21207	66.5	33.5
**24/08/21**	Tue	34	15011	6500	21511	69.8	30.2
**27/08/21**	Fri	35	17159	7600	24759	69.3	30.7
**31/08/21**	Tue	35	14693	6400	21093	69.7	30.3
**03/09/21**	Fri	36	16805	7600	24405	68.9	31.1
**07/09/21**	Tue	36	17915	7500	25415	70.5	29.5
**10/09/21**	Fri	37	16136	7600	23736	68.0	32.0
**14/09/21**	Tue	37	19782	7600	27382	72.2	27.8
**17/09/21**	Fri	38	17969	8300	26269	68.4	31.6
**21/09/21**	Tue	38	21134	8300	29434	71.8	28.2
**24/09/21**	Fri	39	19652	9400	29052	67.6	32.4
**28/09/21**	Tue	39	20715	8200	28915	71.6	28.4
**01/10/21**	Fri	40	19071	9500	28571	66.7	33.3
**05/10/21**	Tue	40	29196	14400	43596	67.0	33.0

**08/10/21**	Fri	41	19649	9800	29449	66.7	33.3
**12/10/21**	Tue	41	12898	7000	19898	64.8	35.2

*** Flies released on these dates were marked with alternated colours and used to calculate Probability of Daily Survival (PDS), Average Life Expectancy (ALE), and Half-Life (HL).**

Table S4. Summary statistics, best fit values, 95% confidence intervals, goodness of fit and normality tests of the three releases of sterile male *D. suzukii* conducted as part of the longevity experiment at the SIT treated site.

	**Replicate / Colour**		
	**Replica 1 / Blue**	**Replica 2 / Red**	**Replica 3 / Orange**
**Best-fit values**			
**Y-Intercept (±95% CI)**	2.26 (1.97 to 2.55)	2.28 (1.88 to 2.68)	2.25 (2.02 to 2.48)
**Slope (±95% CI)**	-0.09 (-0.12 to -0.06)	-0.10 (-0.14 to -0.05)	-0.10 (-0.12 to -0.07)
**Goodness of Fit**			
**Degrees of Freedom**	10	10	10
**R squared**	0.80	0.71	0.89
**Adjusted R squared**	0.78	0.68	0.87
**Sum of Squares**	0.28	0.54	0.18
**Sy.x**	0.17	0.23	0.14
**RMSE**	0.16	0.22	0.13
**Normality of Residuals**			
**Shapiro-Wilk (W)**	0.95	0.91	0.97
**P value**	0.57	0.24	0.86
**Passed normality test (alpha=0.05)?**	Yes	Yes	Yes
**P value summary**	ns	ns	ns

Table S5. Fertility of wild female *D. suzukii* collected from the control and SIT treated sites from week 30 to 41. Table shows the number of females collected (control sites combined), number of eggs laid, number of pupae generated from those eggs and the estimated fecundity (number of eggs per female per 24 hour laying period) and fertility (percentage of eggs that produce pupae). Colour-coded cells highlight changes in fecundity and fertility over time, with darker colours for value increases.

	Control sites					SIT treated site				
**Week**	**No. wild females**	**No. eggs**	**No. pupae**	**Fecundity**	**Fertility (%)**	**No. wild females**	**No. eggs**	**No. pupae**	**Fecundity**	**Fertility (%)**
**30**	2	22	20	11.00	90.91	11	214	136	19.45	36.71
**31**	8	52	42	6.50	75.40	29	193	58	6.66	42.23
**32**	18	151	43	8.39	32.76	25	206	88	8.24	56.44
**33**	12	224	193	18.67	68.88	23	193	75	8.39	43.75
**34**	8	0	0	0.00	_	25	246	210	9.84	83.09
**35**	13	237	177	18.23	72.58	38	706	410	18.58	60.56
**36**	22	155	142	7.05	92.48	90	1098	719	12.20	65.94
**37**	39	760	511	19.49	74.58	31	582	393	18.77	62.04
**38**	90	359	304	3.99	79.98	220	753	525	3.42	69.31
**39**	66	115	55	1.74	59.28	244	826	504	3.39	57.68

**40**	71	0	0	0.00	_	383	39	21	0.10	48.99
**41**	71	1	1	0.01	100.00	265	21	6	0.08	57.41
